# Microcredentials training in pharmacy practice and education: an exploratory study of its viability and pharmacists’ professional needs

**DOI:** 10.1186/s12909-022-03341-7

**Published:** 2022-04-29

**Authors:** Peggy Lok, Kebede Beyene, Ahmed Awaisu, David Woods, Nadir Kheir

**Affiliations:** 1 Faculty of Medical and Health Sciences, The University of Auckland, Auckland, New Zealand; 2 College of Pharmacy, QU Health, Qatar University, Doha, Qatar; 3 School of Pharmacy, Otago University, Dunedin, New Zealand; 4 College of Pharmacy and Health Sciences, Ajman University, Ajman, United Arab Emirates

**Keywords:** Microcredentials, Nano-credentials, Digital-badges, mini-badges, Pharmacy

## Abstract

**Background:**

Microcredentials (MCs) are short courses that certify/recognise an individual’s achievement of specific skills or knowledge. Schools of pharmacy could be well-placed to contribute to the continuing professional development (CPD) of pharmacists through the inclusion of MCs training in their programs. This study aimed to explore pharmacy professionals’ views on the need and viability of MC courses globally.

**Methods:**

Eleven semi-structured telephone interviews were conducted with pharmacy practitioners, policymakers, and academics across seven countries. The participants were selected using purposive sampling to explore information from varying pharmacy disciplines. Interviews were audio-recorded, transcribed verbatim, and analysed using a general inductive approach.

**Results:**

Participants regarded MCs in pharmacy as an innovative idea, well-suited to the increasingly technology-driven world. They believe MCs provide easily accessible means of skills and knowledge acquisition that fulfils the needs of the pharmacy profession. MCs were also perceived as an alternative pathway of meeting the requirements of traditional CPD programmes. Many participants believe universities are well-suited to provide MCs; however, numerous challenges such as recognition, time and resources have been identified as potential barriers to enrolment and implementation.

**Conclusions:**

This study provides an insight into the views of pharmacy practitioners and academics on MCs, and their potential utility in pharmacy education and practice. The findings should help in the development of MCs that could be utilised by pharmacy practitioners around the world for CPD purposes. This study comes at a time when alternative models of teaching and learning are being explored as a direct result of the COVID-19 pandemic.

## Background

The pace of change in healthcare has accelerated rapidly in recent decades, and as a result, healthcare professionals found themselves to always have to commit to lifelong learning (LLL) through continuing education (CE) and continuing professional development (CPD) programs. Being able to adapt to the evolving healthcare needs is an integral part of providing safe and effective healthcare. The change in the healthcare setting is apparent in pharmacy, where new guidelines for the treatment of diseases and numerous new drugs as well as cognitive services are introduced periodically to provide more safe and effective interventions. Most healthcare providers hold higher education qualifications, such as diplomas or bachelors degree. However, in recent years, new ways of acquiring skills, knowledge, and credentials are emerging and engaging millions of learners. These ‘alternative credentials’ include microcredentials (MCs) and industry-recognised certificates. MCs are learning activities consisting of short, self-paced courses that provide opportunities to develop new and existing skills, competencies and knowledge specific to a certain profession [1]. A digital badge is awarded upon completion of a course [2]. MCs are labelled differently across course providers, such as digital badges (DBs), nano-credentials, and nano-degrees. These are associated with MCs and all validate the mastery of a defined set of knowledge, skills or competencies of an individual in any field of specialization [3]. MCs were first introduced in 2010 at a conference in Barcelona (Spain) and have since garnered international attention as a potentially positive disruption across all levels of education [4].

MCs address the gap for those who need continuous skills and knowledge updates, such as those working in a highly transformative healthcare setting [5]. By breaking learning into smaller sections, MCs allow busy professionals to continue learning, whilst quantifying and documenting their development in a verified manner [6]. Furthermore, MCs provide a pathway towards specialization [6], for example, offering services in anticoagulation and anticonvulsant clinics [7]. Previously, to perform these services, post-licensure education, certification and training must be undertaken [8]**,** but MCs can be introduced as a form of credentialed learning, allowing increased learning access to these specialty services [9]. Until now, there has been limited utilisation of MCs within the pharmacy profession, but there appears to be a scope for implementing them in the future. The pharmacist’s expanding roles, a growing trend towards specialisation, and the need for continuous learning and mastery of skill sets has catalysed the interest in add-on credentials [10].

As MCs are in their infant stages of development globally, there are significant challenges before they can be implemented. These include, but not limited to, establishing their credibility/authority, achieving widespread acceptance by learners and professional bodies, integration into existing workflows, and barriers related to time and resources [11–13]. As MCs are implemented, considerable research will be required to investigate their full impact on the workforce and health outcomes, education providers and the healthcare professionals and to explore how MCs can be further improved to enhance their effectiveness. As MCs are a new concept and currently lack informative data, drawing firm conclusions as to their value and viability will be difficult. As a result, in-depth qualitative studies capturing multi-perspective from a range of country representatives from the pharmacy profession regarding the implementation of MC training in pharmacy practice are warranted. This study aimed to explore the perspectives of pharmacy professionals from both developing and developed countries on the need for and the viability of MC courses.

## Methods

### Study design

This was an exploratory qualitative study utilizing semi-structured telephone interviews with a purposively selected sample of pharmacy academics and practitioners from seven developed and developing countries. Ethics approval was obtained from the University of Auckland Human Participants Ethics Committee (reference number 022828, dated 16/04/2019).

### Sample recruitment

A combination of snowballing and maximum variation sampling methods were employed to recruit potential participants from seven countries: Egypt, Brazil, Kuwait, China, New Zealand, UK, and Japan. Individuals invited to take part in the project were purposively chosen based on familiarity with their contributions in professional practice, education, and/or leadership in their respective countries. Given the limited number of participants from each country, data saturation was not sought, nor was it necessary as the focus was collecting information that was broad enough to sufficiently describe a range of perspectives on MCs within and across countries. Interviews were conducted with a range of pharmacy professionals, including academic, hospital, and community pharmacists. Participant information sheets and consent forms were emailed to all potential participants, and all interviewees provided written informed consent before the interview. No compensation was provided for participation in the study.

### Data collection

Data were collected using one-to-one semi-structured interviews to allow flexibility. All interviews were conducted in English, using a questionnaire root that was prepared a priori to help remind the interviewees of relevant and important questions**.** The guide was developed based on literature review and the research team’s experience and it covered a range of topics related to MCs. It was reviewed by a subject matter expert and tailored to participants’ experiences and practice settings. The interview guide was also piloted to ensure clarity of the questions and to optimise the flow of the interviews. Overall, the participants had the opportunity to speak at length about their views on MCs, current needs for MCs and gaps in skills in their respective countries, viability, challenges, and opportunities for MC training as well as ideas on how to better offer MCs in the context of their country. To ensure participants understood the purpose of the study, all participants were provided with a general definition of MCs and were instructed to focus on MCs in pharmacy settings. Participant responses were probed for clarification or to provide more depth. Demographic data, including gender, practice setting type and location, work experience and qualifications, were collected from each participant. The interviews were conducted between June and September 2019. The interviews were audio-recorded and completed via face-to-face, phone or Skype/Zoom calls and lasted from 30 to 45 min. Brief field notes were also taken during the interviews. Participants were informed that they could ask to stop the recording at any time. No compensation was provided for participation in the study.

### Data analysis

All interviews were audio-recorded and transcribed verbatim. The transcripts were printed out and de-identified to ensure anonymity. The field notes were also typed up and organised for analysis. The data were analysed using a general inductive approach (GIA) [14]. This approach allows the research findings to emerge from the dominant themes inherent in the raw data and helps to condense extensive text data into a brief summary and to establish clear links between the research objectives and findings.

Initially, the transcripts were reviewed in their entirety whilst listening to the audio-recordings to gain a sense of the data before coding of the data began. Data coding was started after all data collection was completed. The initial coding framework was developed based on the main questions in the interview guide. Then, three transcripts were reviewed and discussed among all authors to identify emerging themes, and the coding framework was revised and expanded. The revised coding framework was applied to additional transcripts to further refine and add themes. After that, five members of the research team coded the remaining transcripts using the revised coding framework. The research team met regularly to discuss emerging themes and resolve coding discrepancies. Instead of line-by-line coding, only sections of the data relevant to the research objectives were coded. If a section of the data had multiple meanings it was coded into two or more categories. After relevant texts of all interview transcripts were linked to appropriate codes, a more focused coding was undertaken in order to identify themes, patterns and relationships emerging across the data. Quotes in each theme were collected and interpreted by coders. When this process was conducted for each transcript, patterns and similarities/differences were identified and further analysed, allowing the researcher to draw critical insights. Attention was also paid to the data that did not fit into the emerging patterns and relationships to explain outlying data or negative cases. Mind maps were used to systematically organise emerging themes and to visualise relationships between themes [15]. Finally, the themes were grouped into fewer overarching themes and sub-themes. It is important to note that coding and themes identification occurred as an iterative process.

To enhance rigour and trustworthiness, multiple strategies were employed during analysis [16]. The data were coded and analysed by multiple individuals. Study findings were presented at departmental seminars and evaluated by peers, and the research team members have many years of qualitative research experience.

## Results

**Fig. 1:**
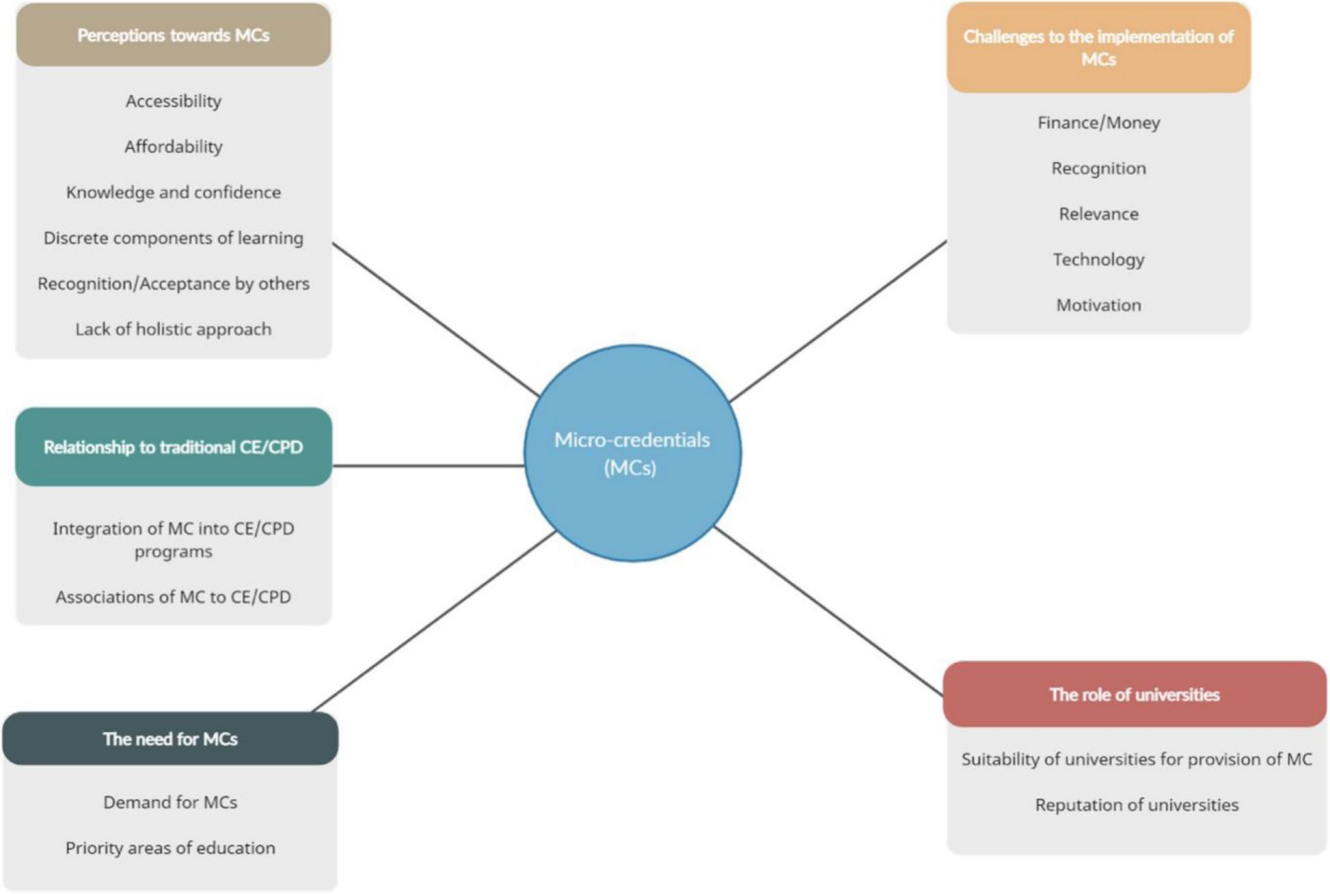
Conceptual map

Between 1 April and 30 May 2019, pharmacy professionals from different countries were contacted via email and invited to take part in the study. Of the 16 individuals invited, 11 accepted the invitation. As shown in Table 1, of the 11 participants interviewed, three worked full-time in academia, one in industry, two in community pharmacy, three in hospitals, and two in government regulatory affairs. Five themes and several categories were identified from the thematic content analyses. These are summarised in Fig. 1 and in the following sections.

**Table 1:** Participants’ characteristics

Participant	Current role/occupation	Country
**1**	Hospital pharmacist	New Zealand
**2**	Pharmaceutical policy	New Zealand
**3**	Pharmaceutical policy	New Zealand
**4**	Hospital pharmacist	New Zealand
**5**	Community pharmacist/Academician	Japan
**6**	Academician	Brazil
**7**	Industry pharmacist/Academician	Brazil
**8**	Academician	Kuwait
**9**	Community pharmacist	Egypt
**10**	Hospital pharmacist	United Kingdom
**11**	Academician	Hong Kong

### Perceptions towards microcredentials

We explored the perceptions of participants on the advantages and disadvantages of MCs as compared to the current traditional learning methods such as postgraduate diploma and certificates. Their perceptions are briefly described below.

The perceived advantages of MCs were largely similar across all participants, irrespective of country. One of the recurring advantages was the flexibility in time and location MCs offered. By offering this flexibility and allowing individuals to learn in their own time, participants from all countries suggested MCs could be easily integrated into the busy schedules of pharmacists. For instance, the participant from Hong Kong stated, “*you can go by your own interest, your own time and own budget*”. According to most participants, postgraduate degrees and certificates require commitment for most or all of the year with fixed times for lectures and workshops and in most cases, this is not possible for pharmacists, who usually work full time. In contrast, MCs are shorter courses, allowing flexibility for learning to be undertaken when it suits the individual.

Another advantage identified by most of the interview participants was the discrete components of education MCs offer and how this can be tailored to an individual’s specific needs. One of the participants from New Zealand stated that MCs offer “*more manageable, bite size chunks of learning*”, highlighting the fact that it is easier to fill one’s knowledge gap, without adding too much commitment and extra work. The Kuwait interviewee recognised “*pharmacists always need a boost or refresher of their knowledge and skills*” and MCs offer the added benefit of “*not only addressing particular learning needs but also enabling skills and knowledge enhancement*”. As highlighted by some participants, this is especially important as there are always new medicines and guidelines being developed and in order to provide the best patient care, pharmacists and other healthcare professionals must actively seek out new education to train and upskill themselves in that area.

Many other participants implied MCs have the potential to improve pharmacist’s performance and ability to deliver quality patient care as MCs could help pharmacists to specialize in specific areas, for example, in anticoagulation management or other areas.

The interviewees were also commented on the advantage of using technology as a new learning platform, including the ability to exchange ideas through online forums, discussions and courses. The Brazilian academician envisioned MCs “*to be the future*” of education and training and that it might become “*the standard for this generation*”. Similarly, the pharmacist from Egypt commented that MCs are well suited to such a “*digital age*” where the world is becoming increasingly technology-oriented. As stated by participants, many newly registered pharmacists grew up alongside technology and would find this method of learning to be more suited and efficient compared to the traditional approaches. However, some participants had concerns about MCs and the increasing reliance on technology. One concern raised by many of the participants was the potential lack of recognition or acceptance of MCs by potential employers and other HCPs, as MCs are a relatively new concept. However, one of the New Zealand hospital pharmacists pointed out that the “*digital badging feature of MCs allows [others] to see what you’ve done, how you’ve done it and how you’re assessed, which can be added onto your CV*,” thus allowing one’s skill and scope of knowledge to be more transparent to others, which is deemed useful even if others were not familiar with MCs.

MCs offer very specific and narrow topics and this was perceived as a disadvantage by the pharmacist from Egypt, “*I think the only disadvantage comes from the name itself. It’s a microdose. A very small dose. And to get good knowledge from a small dose, you need a lot of them. You also need to continue on having them so you can build up good knowledge - one or two is not enough*.” These views were in agreement with one of the NZ hospital pharmacists, who stated, “*postgraduate courses are better at moulding a lot of information together*” and that “*[MCs] do not allow us to think about the bigger picture*.”

### Microcredentials and CPD/CE

Considering the agreed description of MCs, participants were asked if they considered MCs as another form of CPD/CE within the healthcare setting. The academic participant from Brazil believed MCs were well-suited to the demands of the new generation, who “*want things to happen right here, right now*”. The participant goes on to justify why these short courses are easily accessible through mobile devices, allowing them to complete their CE/CPD learning anywhere and anytime, and thus, it “*matches the new generation of how things are happening*.” In that sense, MCs and traditional CE can work together to make education more accessible and feasible. Other participants had similar views, especially because technology has now “*heavily integrated into our lives*” and MCs will be a great way to “*dominate the future*” of education.

The participant from Japan explained that their CE programme (e-learning) was currently based on a point system, while MCs operate on an innovative badging system. The participant from Egypt also made a similar observation, suggesting that MCs allow certificates/badges to “*build upon each other*” which has the added benefit of further motivating the user. However, they believed if MCs could offer a point system to allow pharmacists to fulfil the traditional CPD/CE requirements, users can be more selective of which MCs to do for their own interest and which ones for CPD/CE. Other interviewees remained more cautious when linking the possibility of integrating MCs to traditional CPD/CE programmes. For instance, the Hong Kong interviewee suggested that “*the design of MCs, the specific learning objectives and the evaluation required to assess whether a candidate may receive that credential must be robust enough to ensure they match traditional CE/CPD programmes.*”

One of the hospital pharmacists from NZ held slightly different views. This participant was unsatisfied with the current CE/CPD programmes offered in NZ by the major organisations, suggesting they needed “*a major review*.” They believe the current programmes did not offer as much learning progression as they can, which is where MCs can fill in that gap. Many other participants mentioned that the traditional programmes were often expensive, making MCs more economically favourable. However, the other NZ participants thought MCs appear to be insufficient for meeting CPD/CE requirements and may negatively affect future learning opportunities. MCs appeared to be an inadequate medium to allow one to demonstrate their ability of being able to complete skills to a *“repeatable standard on an ongoing basis*” which is required for a pharmacist to demonstrate their competence in a certain skill. There were also speculations that excessive micro-credentialing can hinder pharmacists from “*progressing along to get those postgraduate qualifications*” as part of their LLL and it will be a “*sad, unintended consequence*.”

### Microcredentials and the role of academia

Participants were generally supportive to the role of universities delivering MC courses, although their views on the level of commitment from universities varied.

Both participants from Brazil expressed similar views, stating that universities have a role in providing MCs. The interviewees also implied that widespread adoption of MCs will be inevitable and “*there’s no way you can run from MCs as they will become even more and more popular form of education*”. Their main concern was around whether their public or private university would be able to do this. There were concerns that MC courses would only be viable in private universities which could prove to be an issue as both believed that the country’s public universities would provide better quality MC courses, but they might not have the funding and resources. Interviewees from Japan, Egypt and Kuwait shared similar perspectives on the role universities can play for the implementation of MC courses. Universities in Japan already have a system providing ongoing e-learning courses for pharmacists and the addition of MC courses provides “*plenty of knowledge*” and will be highly sought after. In contrast, the UK pharmacist believed as long as high quality structured courses were delivered and strict academic integrity assessment was in place, other accredited providers (not only universities) could provide MC courses. The Hong Kong participant shared a slightly similar view. He recognised that limiting the design and implementation of MCs to universities will be insufficient and inadequate, and stressed the importance of striving to collaborate with practitioners in the community to “*enhance educational content*” so it is a better reflection of “*what is needed at the community*”..

There was consistency where the reputation of the university involved in the delivery of MC courses should be considered. For instance, one of the NZ pharmacists working in policy stated “*pharmacists might want to pick a university that has a great global academic reputation*”, especially considering MCs are delivered online and easily accessible to anyone in the world.

### The need for microcredentials

The topics and needs of MCs were investigated to gauge the viability of implementing MC and digital badging systems. Most representatives identified a market for MC implementation in their countries, which would benefit the development of individual pharmacists and the profession.

Both pharmacists from Brazil shared similar opinions on the need for MCs. However, as legislation is changing and some pharmacists are being proactive and trying to develop their skills, they believe MCs may help motivate and facilitate this.

The participants from Brazil, Egypt and Kuwait believed MCs would be beneficial to “*decompose large topics into smaller ones*” and develop skills further as they expressed how busy pharmacists are in practice and so MCs would be “*easier to do*” instead of completing post-graduate courses.

There was agreement among the participants where all pharmacists “*should have the motivation to learn new knowledge and skills*” facilitating the development of specialised areas and increasing the pharmacists’ scope of practice, leading to the advancement of the pharmacy profession.

Japan’s participant mentioned how “*many Japanese pharmacists want to go abroad*” and MC expansion across the globe could allow certification and recognition of pharmacists’ skills in other countries. Due to several natural disasters that have taken place in Japan, the representative expressed particular interest in topics involving the supply and management of medicines during natural disasters.

The Hong Kong interviewee explained that the viability of MCs in Hong Kong “*depends on the design and learning objectives*” and was unsure about the difficulty levels MCs could offer and how candidates will be evaluated to obtain the credential.

The UK pharmacist thought if MCs are to be implemented on a global scale, it will be difficult to assess and meet the needs of everyone in different countries as “*various countries have variable practice*”. This may mean that a course in one country may not be relevant to another country.

Views around the need for MCs in NZ were similar and positive across the four participants. Unlike the pharmacists from Japan and Kuwait, one of the NZ pharmacists thought MCs are more suited to pharmacist’s own personal development, rather than changing the pharmacy profession, expressing views on how pharmacists would “*always benefit*” from courses that enhance their professional development. She stated that MCs would allow “*pharmacists to identify gaps in their skills and find training opportunities to fill these gaps*”, developing soft skills such as communication, teamwork, leadership, cultural competence and Te Reo Māori. It will also allow pharmacists to explore the opportunities in areas that are of interest to them but not available in their own workplace.

### Challenges to the implementation of microcredentials

Despite the overall positive views towards MCs, a range of challenges were identified by the study participants. Most challenges proposed can be grouped into sections.

#### Cost

Each participant expressed the challenge of dealing with time and costs to varying extents. Firstly, the cost of MC courses was a prominent discussion by participants from Kuwait, NZ and Japan, emphasising the true cost of MCs are worth considering. This not only includes the cost of the MC course itself but also the cost of setting MCs up, keeping it running and the time spent investing in MCs compared to something else. Having to pay for MCs out-of-pocket were also concerns from many of the participants as most pharmacists consider their salary quite low already.

#### Technology

The type of electronic device, interface, WIFI capabilities and ability to download MCs could be a challenge were some of the technological challenges mentioned by most participants. The interviewees stated that if WIFI is subpar and the MC interface is complicated or underdeveloped, pharmacists are less inclined to participate. According to participants, this is particularly a challenge in developing countries where there are significant IT infrastructure deficits.

#### Legislation and regulation

MCs are a new concept and regulation of these courses are required. DBs in most countries currently do not have any legislation protecting or implementing them; however, for MCs to be a form of recognised CPD, it was stated by participants that legislations and regulations are a must. Legislations also provide reassurance the education they’re receiving are current and standardised. Some countries do not have any legislation for CE, meaning MC courses may mean little to nothing for pharmacists. Participants believed that the uptake of MC courses in such countries would be dependent on the individual and how motivated they were to upskill and train themself. As has been described above, potential lack of recognition of MCs by employers, professional associations, and other authorities were also mentioned by most participants as the other challenge for MCs programs.

#### Motivation and application

A common, perceived challenge among the participants was the motivation of the pharmacist in undertaking these MC courses. There was further elaboration regarding the selection of MC courses as they believed if courses weren’t chosen carefully and/or they were irrelevant to pharmacists, there would be very few people attending. This includes applicability to local practice and/or guidelines. While there can be vast differences in the way pharmacists practice in different countries, there are also overlapping similarities. Therefore, according to participants, it would be important to focus on back-to-basic topics and skills globally, with more specific courses aimed at each country to match their practice and guidelines.

## Discussion

This study addressed a few major issues related to MCs including perceptions towards MCs and its relationship with CPD/CE, the role of universities with regards to the provision of MC courses, the need for such courses, and, finally, foreseeable challenges with regards to its implementation. Recruiting participants from a range of countries was meant to provide us with a wider cross-section through sampling countries (and practices) that would allow us to compare and contrast possibilities and challenges as expressed by our selected individuals, and how these differ between countries. We feel this information would be valuable to readers in different locations in the world.

Most of the participants acknowledged the unique features MCs had to offer, such as being less costly, more flexible and easily accessible. This was in line with findings from the literature search that was conducted which explored MCs and its use in other professions [17–23]. Participants expressed interest in utilising MC courses tailored to their own field in pharmacy which provides the opportunity to acquire the required level of skills necessary to specialise within a certain area. Practitioners may be attracted to this trainee-centered approach, which would allow them to better invest their time to fulfil their own learning and training needs. Equally attractive to participants was that MCs offer the chance to pursue topics according to their own interests and desires which can be interpreted as the freedom and power to direct their own professional development. Indeed, the literature on MCs suggested these courses helped students’ exercise full autonomy while identifying the courses appropriate to their needs in various settings [24].

The interest expressed towards MCs shows pharmacists wanting to improve their professional skills, provided they could integrate the training easily into their daily schedules. This has been found in earlier research where a survey revealed that 34% (*n* = 1239) of PharmD students in the US exhibited the desire to pursue additional training after graduation [25]. Additionally, throughout the last few years there has been a clear shift of pharmacists from a traditional dispensing role to a more clinical patient-centered role [26]. The shift to a more clinical role requires synthesising and linking the pharmacist’s clinical knowledge together. As some pharmacists may have been in the traditional dispensing role for a long time, small MC courses may be what is required to boost the pharmacist’s confidence and offer an accredited set of skills and knowledge to fulfil the growing expectations in their profession.

Doubts around the recognition and acceptance by the relevant stakeholders were expressed. MCs are a relatively new concept, and many people are still unfamiliar with what they are and how they can help a pharmacist progress. However, some participants in this study argued that digital badging (DB) linked to MCs offer enough transparency and are self-explanatory by nature. This is in accordance with the current literature, which agrees DBs are an enriched symbol that houses valuable information with regards to one’s skills and accomplishments [27, 28]. Similar concerns were also reflected in previous studies that explored the effect of DB systems amongst the workforce [29–31]. Thus, lack of recognition and acceptance is a potential issue that may be remedied over time, as MCs begin to flourish more within the healthcare setting and subsequently raise more awareness.

Other notable challenge perceived by participants related to the nature of MC courses being short and discrete components of learning. The growing complexity of patient’s medical health needs have been highlighted by numerous studies [32–34]. As people live longer, healthcare professionals are dealing with increasingly complex cases and MCs may lack the ability to reflect real life practice. Some participants questioned if MCs would be able to approach learning in an integrated and holistic manner. MCs might be more suited to address basic and foundational skills/knowledge required in pharmacy but whether they will be able to address deeper content remains an area to be explored through research. It is possible to create MC courses dedicated to specialised and complex cases, however, such courses will require specialist knowledge and will only target a small audience as it may not be relevant to most healthcare professionals in their everyday practice.

Overall, participants seemed to advocate for the idea that MCs could offer an alternative pathway to fulfilling CE/CPD requirements, with the added benefit of enhanced flexibility, accessibility, and convenience. This comes from the participant’s personal experiences with the more traditional ways of fulfilling CE/CPD requirements such as via conferences, seminars, workshops - all of which demand physical attendance and substantial time and money. Current literature recognises such factors are major barriers toward the participation of these activities [35, 36]. MCs are perceived as having the potential to dismantle some of these barriers. As mentioned in the literature, MCs break learning into small chunks, allowing busy professionals to continue learning, whilst quantifying and documenting their development in a verified manner [6]. Our results support this finding and it is a highly attractive quality for the pharmacy profession. Research did show how the underlying principles of MCs align closely with the core values of CE/CPD in healthcare, i.e. self-directed, based on learning needs and having defined outcomes [37]. Seeing traditional CE/CPD are deemed ineffective, it may be worthwhile to trial MCs [38]. There is also concern some CE/CPD providers are unable to identify and respond to individual pharmacy needs [37]. Participants generally shared the insight that MCs would enable pharmacists to get the most out of their CE/CPD requirements, making their learning more valuable and applicable to their own practice.

Our study highlighted some concerns around the practical design, quality and assessment of MC courses. Many participants highlighted that if such a system were to be implemented, it must be robust and challenging enough to match the academic standards of traditional CE/CPD programmes. Despite MCs being relatively shorter, it should not compromise the strict academic expectations. A certain degree of apprehension exists with regards to the possibility MCs could prevent pharmacists from pursuing higher education such as postgraduate studies but further research is needed to make this conclusion.

To date, no MCs have been studied within the context of pharmacy CPD/CE although they appear promising in other settings such as the teaching and medical field [17, 18, 23]. This study reveals practitioners see MCs as a viable option to help pharmacists fulfil their CE/CPD requirements. These results also confirm the ideas present in previous papers, which state MCs can address the gap for those who need continuous skill/knowledge updates, such as those who work in a highly transformative healthcare setting [5].

There was consensus that universities are well placed to create and deliver MC courses for the pharmacy profession. A number of MCs are already provided by leading universities, such as MIT and Harvard [39–41]. Participants offered various reasons for their views: universities’ expertise in academia, being a great addition to the training/education they already provide and its reputation in delivering quality education. It was noted the rankings of those that provided such courses were important, suggesting participants placed an emphasis on the prestige and reputation of the provider. Ultimately, this implies the driving force for pursuing MCs is the desire to obtain quality education and such achievements need to be recognised by others.

Other participants built upon the idea by highlighting the need for universities to collaborate with local practitioners to ensure courses reflect current, real life practice. A couple of participants indicated the explicit need for universities to implement such courses for the ongoing training of pharmacists as many of the external organisations have been regarded as less cultivating and operate heavily in the interests of business profit.

Participants also affirmed the idea MCs and its DBs were an innovative way to showcase to peers or other HCPs the skills/knowledge they have. An earlier paper found pharmacists who earned DBs reported elevated feelings of self-worth and increased recognition by other HCPs [42]. Other studies also found DBs a great way to illuminate one’s skills/knowledge for employers. In light of these results, it seems fair to suggest verification/acknowledgement of one’s competencies is highly desirable. Thus, within the context of pharmacy, DBs allow more transparency of the individual pharmacist’s expanding skill sets, translating into better utilisation of their skills and expertise.

In agreement with other research [6, 43, 44], our findings suggest the length of time an MC course takes to complete is a major determinant of whether it will be considered. Although time constraints are well known to researchers [11], the aspects of cost have been largely unexplored. Different countries offer pharmacists different salaries, therefore cost is relative to each situation and depends on factors such as who will pay for the costs, who will fund the courses and who will run the courses. Some pharmacists aren’t required to participate in CE/CPD and for those, any cost associated with the courses may not be popular. Limitations aside, this study revealed the concept of cost is more complex than previous studies suggest [11]. Adding directly to cost is technology, which is expected by participants to be advanced and affordable with MC courses. Another point to consider is if participants do not have the technology at home which places them at an instant disadvantage and provides even greater time constraints for completing these MC courses.

Participants described individual motivation as a potential challenge. This stems from the worry of the time and effort spent pursuing these courses which may not be recognised by others and the fear of exploring something beyond traditional learning methods. Considerable effort seems to be required to promote and advertise MCs to pharmacy practitioners and show the positive impact it can have on one’s career. In line with other research, there is concern regarding the lack of applicability of MCs to each and every practicing environment [43], ultimately hinting each MC must be tailored to that specific country to provide value that can serve the profession.

These findings advance our understanding and provide further insight into the need for legislation and regulation around MC courses so standards of care are kept and competencies are made. Previous studies have discussed the fact that legislation is more difficult to change than regulations [45].

Participants suggested formal recognition of MCs so they could contribute to the development of pharmacy practice and to the advancement of the profession. This aligns with finding from earlier studies outlining that addressed the issue of recognition and relevance of MC courses especially when compared to more traditional degrees [46, 47].

### Limitations and strengths of the study

We were able to recruit only limited number of participants from each country. Ideally there would have been more than one interviewee representing each country, in order to fully capture the views unique to that practice environment. However, the heterogeneity of the pool of countries (i.e. each country with unique practice environments and perspectives) made data saturation un-achievable. A few of the countries such as Japan and Egypt only had one interviewee, meaning there was greater risk of personal bias and their responses may not have been an accurate reflection of their country. The study participants views are also less transferable to other practitioners working in a different pharmacy discipline. Furthermore, a few participants withdrew from the study, including 1 from Canada and 3 from NZ (mainly due to time constraints), ultimately reducing the pool of countries and the number of representatives. There were also more interviewees from New Zealand compared to the rest of the countries. However, at the time of the study, New Zealand had been embarking on active plans to start MC courses in one of its Universities, and the study was of special interest to the academic circles and other stakeholders there.

## Conclusions

To our knowledge, this is the first study to explore MC needs in the pharmacy setting and thus contributes valuable insight into this unexplored territory. These insights can be used to guide the development of appropriate MC courses for pharmacy practitioners. Also, this study comes at a time where schools and academic institutions globally are looking for alternative forms of education because of the impact caused by the CVID-19 pandemic. The study was conducted before the pandemic, therefore no specific questions relating to COVID-19 were used. As MCs are a relatively nascent enterprise, considerable challenges lay ahead of its implementation. These revolve around time/money, legislation/regulation and the potential recognition by stakeholders. Nevertheless, a myriad of subject areas were identified to be of interest, including assertive communication, leadership, nutrition, and pharmacy business management. Drawing on the results of this study, there are reasons to think the launch of a suite of pharmacy related MC courses will be a worthwhile enterprise and that it is more likely to gain interest and support than not. However, further qualitative and quantitative research is warranted to narrow down the exact topics that will be of universal interest, to ensure there is maximum utilisation of such MC courses when provided.

## Data Availability

The author confirms that all data generated or analyzed during this study are included in this published article.

## Abbreviations

MC: Microcredentials
CE: Continuing education
CPD: Continuing professional development
